# Investigating the Microstructural and Textural Properties of Cookies Using Plant-Based Bigel as an Alternative to Commercial Solid Fat

**DOI:** 10.3390/gels11080571

**Published:** 2025-07-23

**Authors:** Ingrid Contardo, Sonia Millao, Eduardo Morales, Mónica Rubilar, Marcela Quilaqueo

**Affiliations:** 1 Biopolymer Research & Engineering Laboratory (BiopREL), School of Nutrition and Dietetics, Faculty of Medicine, Universidad de los Andes, Chile, Monseñor Álvaro del Portillo 12.455, Las Condes, Santiago 7620086, Chile; 2 Centro de Investigación e Innovación Biomédica (CIIB), Universidad de los Andes, Chile, Monseñor Álvaro del Portillo 12.455, Las Condes, Santiago 7620086, Chile; 3 Department of Chemical Engineering, Faculty of Engineering and Science, Universidad de La Frontera, Temuco 4780000, Chile; sonia.millao@gmail.com (S.M.); eduardo.morales@ufrontera.cl (E.M.); monica.rubilar@ufrontera.cl (M.R.); 4 Scientific and Technological Bioresource Nucleus, BIOREN, Universidad de La Frontera, Avenida Francisco Salazar 01145, Temuco 4811230, Chile

**Keywords:** canola oil, carnauba wax, Arabic gum, oleogel, hydrogel, hybrid gel, porosity, texture

## Abstract

In response to the growing demand for improving the nutritional profile of widely consumed products, such as cookies, there has been an increasing interest in fat replacers that preserve sensory attributes and have a more positive health effect. Among the novel fat replacement strategies, the incorporation of bigels into food formulations has been studied; however, the impact of Arabic gum hydrogel-based bigels on microstructural properties and their correlation with the texture and quality of bakery products remains underexplored. In this study, cookies were formulated using a plant-based bigel (canola oil-carnauba wax oleogel mixed with Arabic gum hydrogel) as a fat substitute, and their microstructural, textural, and quality parameters were compared with those of commercial butter-based cookies. Compared to butter (firmness of 29,102 g, spreadability of 59,624 g∙s, and adhesiveness of 2282 g), bigel exhibited a softer (firmness of 576 g), more spreadable (spreadability of 457 g∙s), and less adhesive texture (adhesiveness of 136 g), while its rheological properties showed similar behavior but at a lower magnitude. Bigel exhibited high thermal stability and good elastic and thixotropic behaviors, indicating reversible structural breakdown and recovery. Cookies prepared with bigels instead of butter exhibited a similar proximate composition, with a slight increase in lipid content (11.7%). The physical dimensions and density were similar across the formulations. However, the microstructural analysis revealed differences when bigels were incorporated into cookies, reducing porosity (55%) and increasing the mean pore size (1781 µm); in contrast, mean wall thickness remained unaffected. Despite these structural modifications, the potential of bigels as viable and nutritionally enhanced substitutes for conventional fats in bakery products was demonstrated.

## 1. Introduction

Bakery and confectionery products are increasingly recognized as essential components of modern diets, driven by their convenience, sensory appeal, and versatility. These products are widely consumed across all age groups because they provide quick and accessible energy sources. Among bakery products, cookies are classic food items that are readily consumable, reasonably priced, and practical snacks. Traditionally, bakery items, including cookies, have been classified as primary sources of carbohydrates, particularly sugars and fats, and are often of lower nutritional value. With fat content reaching up to 40%, these products achieve desirable textural and sensory characteristics that contribute to their popularity; however, this comes at the expense of energy-dense food choices [1,2].

The primary fats utilized in the food industry for cookie production include butter, margarine, and shortening, which are high in calories and saturated fatty acids, and margarine with potential trans fatty acid content. However, the growing awareness of health, sustainability, and consumer preferences is driving significant changes in the industry. Excessive consumption of saturated and trans fatty acids has been linked to an increased risk of obesity, diabetes, and coronary heart disease. Consequently, the US Food and Drug Administration excluded partially hydrogenated vegetable fats, the primary source of industrial trans fatty acids, from the “generally recognized as safe” (GRAS) list in June 2018. Replacing trans fatty acids with healthier oils and fats rich in mono- and polyunsaturated fatty acids (MUFAs and PUFAs, respectively) has been shown to reduce the risk of cardiovascular diseases. In this context, developing commercial fats that utilize vegetable oils with a higher proportion of PUFA while maintaining low levels of saturated fatty acids (SFA) and being free of trans fatty acids remains a significant challenge. For example, canola oil contains only 7% SFA, but 62% MUFA, mainly oleic acid, and 32% PUFA, which includes two essential fatty acids: linoleic acid (omega-6) and linolenic acid (omega-3). Achieving this balance is critical because a low saturated fatty acid content can compromise the techno-functional properties of these fats [1,3,4]. Cold-pressed canola oil is nutritionally superior to refined canola oil because of its high content of omega-3 fatty acids, tocopherols, phytosterols, and polyphenols. These components contribute to its health benefits, including improved lipid metabolism, reduced oxidative stress, and anti-inflammatory effects, making it a better choice from a nutritional perspective [5,6].

A promising strategy for developing healthier solid fats involves the transformation of edible oils rich in unsaturated fatty acids through oleogelation, a non-triglyceride oil-structuring technique. Oleogels provide a potential solution by converting liquid oils into gel-like systems that mimic the functional properties of saturated fats, while incorporating beneficial MUFA and PUFA. However, oleogels maintain the caloric density of fat. Recently, bigels, composite matrices formed by combining two gelled phases, an oleogel (apolar solvent-based) and a hydrogel (polar solvent-based), offer expanded opportunities to tailor fat structures. These systems have diverse applications in the food and pharmaceutical industries. In food matrices, bigels could make it possible to customize fat properties while reducing caloric density compared to traditional fats, depending on the matrix design, presenting a valuable innovation in developing healthier food products [2,7].

Bigel matrices have recently demonstrated significant potential as fat substitutes for cookies. For instance, bigels composed of beeswax–canola oil oleogel and carboxymethylcellulose or sodium alginate hydrogel in a 50:50 oleogel-to-hydrogel ratio produced cookies with hardness comparable to that of the original product. However, specific physical properties, such as fracturability and geometry, are affected [8]. Further research is needed to clarify the interactions between bigels and cookie ingredients to optimize bigel properties. This advancement could facilitate the production of healthier food products with an improved fatty acid profile while preserving the sensory and physical attributes of the original product.

Bigel properties can be tailored by modifying their composition, such as the choice of structuring agents and oleogel-to-hydrogel ratio, as well as processing conditions, including mixing temperature and speed [7]. Carnauba wax (CW), a natural wax obtained from the leaves of the Brazilian palm *Copernicia prunifera*, has been extensively studied as an oleogel in combination with various liquid oils, including insect oil, sunflower oil, and hemp seed oil, and has shown promise as an adequate fat substitute in cookies [9,10,11]. Conversely, Arabic gum (AG), a natural gum extracted from the acacia tree, exhibits low viscosity and high solubility, offering good stabilizing and emulsifying properties; furthermore, AG is a source of dietary fiber and has prebiotic effects, making it a valuable component in functional food formulations [12]. The preparation of bigels from CW/canola oil oleogel and AG hydrogel has been reported previously, demonstrating the formation of viscoelastic matrices with a softer and spreadable texture, as well as thermal resistance at temperatures up to at least 50 °C [13]. However, these matrices have yet to be incorporated into food as a substitute for butter to investigate a healthier alternative to food ingredients.

This study hypothesized that incorporating plant-based bigels as a substitute for commercial butter in cookie formulations would produce cookies with quality, microstructure, and textural properties comparable to those prepared with butter. A bigel was prepared by mixing a CW/canola oil-based oleogel with an AG-based hydrogel; the selection of the process conditions to prepare the bigel was based on a previous study reported by Quilaqueo et al. [13], seeking to obtain a semi-solid matrix with a high solvent (oil and water) binding capacity. A commercial butter was used as the control. The knowledge acquired will help expand the application of bigels in the rational design of healthy and low-fat baked food products.

## 2. Results and Discussion

### 2.1. Characterization of the Bigel and Commercial Butter

**Figure 1:**
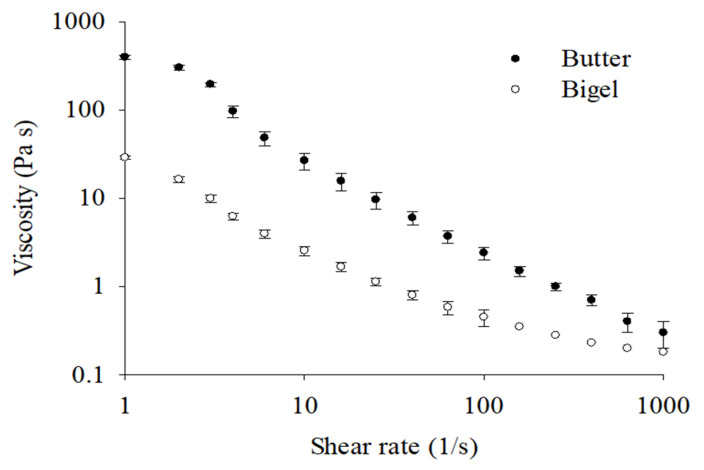
Apparent viscosity (Pa·s) of butter and bigel over a shear rate range of 1–1000 (1/s).

The mechanical properties of bigels are critical for their specific applications. A texture analysis revealed that the textural parameters of bigel differed from those of butter (Table 1). The firmness of butter was 50 times higher than that of bigel. The spreadability, which indicates how easily the sample can be spread into a thin, even layer, was 130 times higher for butter than for bigel. The adhesiveness of butter was 16 times higher than that of bigel. With 52% SFA, the butter’s composition provided a structure with strong textural characteristics. However, the rheological patterns of bigel and butter are similar, although their magnitudes are different. The apparent viscosities of bigel and butter exhibited pseudoplastic behavior, with their values decreasing as the shear rate increased (Figure 1). However, the viscosity of butter was higher than that of bigel over the entire analyzed range (from 1 to 1000 1/s), which aligned with the high firmness of butter determined by texture analysis. These results are consistent with those of other studies that have established that butter viscosity is higher than that of bigel, which has been attributed to its composition. The viscosity of butter is influenced by its fat content and fatty acid composition, whereas in bigels, the viscosity is affected by the type and concentration of wax and the interactions between the oleogel and hydrogel components. Wax has been shown to produce oleogels with specific rheological properties, including increased hardness [14,15,16].

**Figure 2:**
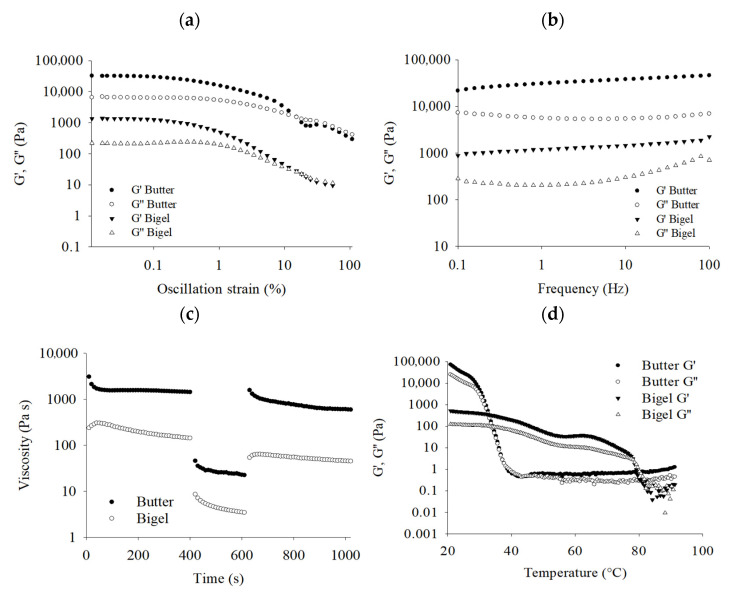
Viscoelastic properties of butter and bigel: strain sweep (**a**), frequency sweep (**b**), thixotropic properties (**c**), and temperature sweep (**d**).

Viscoelastic analyses showed that the oscillation strain could be divided into linear (LVR) and nonlinear (NLVR) viscosity regions (Figure 2a). In the first region (from 0.01 to 0.1%), LVR, G′ was higher than G″ for both samples, indicating a solid-like elastic behavior. As the strain increased, NLVR was observed in the second region (>0.1%), with sharp decreases in G′ and G″. The moduli values of the butter were higher than those of bigel during the entire oscillation strain sweep, suggesting that butter had greater strength. Interestingly, the transition of bigel from elastic (solid-like) to viscous (liquid-like) behavior (the crossover points of the network structure) occurred at an oscillation strain (approximately 10%) similar to that of butter; however, a more progressive transition was observed in bigel. Therefore, a structural breakdown occurred, resulting in a delay. In addition, a greater difference between G′ and G″ was observed for bigel, suggesting that this is a structured system with good gel characteristics. In the frequency sweep conducted in the LVR, the storage modulus (G′) of both samples remained higher than the loss modulus (G″), demonstrating a solid-like behavior (Figure 2b). No crossover point between the moduli was observed, indicating the adequate stability of both bigel and butter. Additionally, although butter displayed higher modulus magnitudes than bigel, the results suggested that bigel has a light dependence on frequency, possibly because of its network structure with stable interactions. In the time sweep evaluation of viscosity (Figure 2c), bigel and butter exhibited thixotropic characteristics with similar patterns but with different magnitudes. Even so, it was observed that bigel and butter had recovered viscosity, maintaining a stable structure that can resist disruption under low (0.1 s^−1^) and high (10 s^−1^) shear rate conditions. These findings are highly relevant for bakery applications that require reversible structural breakdown and recovery, demonstrating the ability to partially reform the damaged structure of the samples studied. Additionally, significant differences were observed between bigel and commercial butter during the temperature sweep (Figure 2d). While the butter’s solid-like structure was lost before 40 °C, the bigel maintained its elastic network at higher temperatures of up to approximately 80 °C, demonstrating that the intermolecular bonding of the bigel was less affected (attributed to stabilized structure, interactions, and the high melting point of CW). The high thermal stability of bigel also indicates that this structured oil might possess good elastic characteristics, such as solid fat, making it more suitable for application in the bakery and confectionery fields. The thermal resistance of bigel could be attributed to the structural characteristics, owing to the synergistic effect between CW and AG. This interaction is mainly governed by physical factors, especially intramolecular and intermolecular hydrogen bonds, which drive the formation of a network in the wax-based oleogel and the gum-based hydrogel. These results are consistent with those reported by other authors. The viscoelastic properties of butter are highly temperature-sensitive, leading to issues with spreadability and oiling off. In contrast, bigels have exhibited more stable viscoelastic properties at different temperatures [16].

**Figure 3:**
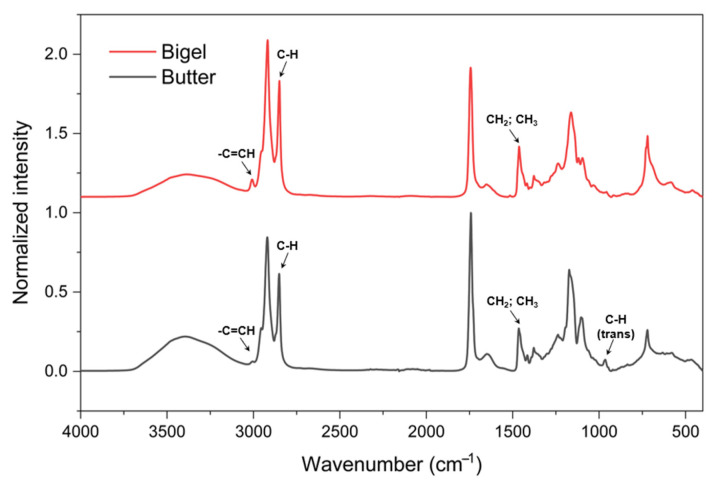
FTIR spectra of bigel and butter.

The FTIR spectroscopic analysis provided a molecular fingerprint of the materials. Figure 3 shows the spectra of the bigel and the butter, indicating their molecular differences. Butter exhibits characteristic lipid peaks, which are similar to those found in the oleogel phase of bigels but differ in the presence of dairy fat-specific peaks. The FTIR spectrum of butter revealed bands associated with milk and fat components. The main bands detected were related to the presence of water, lipids, and proteins, which belong to the functional group region (4000–1500 cm^−1^). The broad absorption band at 3394 cm^−1^ was assigned to the hydroxyl stretching vibration of water (OH). The band related to the stretching of the -C=CH groups of the cis-unsaturated groups reached its maximum height of 3005 cm^−1^ in butter and shifted to 3008 cm^−1^ in the bigel sample. The intensity of this band was greater for bigel, demonstrating the greater presence of cis-unsaturated groups in this structured matrix. The band at 966 cm^−1^ is indicative of trans fatty acids in butter, specifically trans C–H stretching vibrations [17]. The peaks observed at 2900 and 2800 cm^−1^ mainly describe C–H (CH_2_ and CH_3_) stretching vibrations. In the 1800–1000 cm^−1^ region, several absorption bands related to the vibrations of the C-O bond of esters were observed. The strong absorption bands of the C=O group at 1742 cm^−1^ and 1651 cm^−1^ were mainly associated with the -C=O stretching vibrations of acids and esters. The C-O ester band appeared at 1173 cm^−1^. The peak at 1465 cm^−1^ and the next arising from the N–H bending vibration are most likely associated with the amide I and amide II bands of proteins [18,19].

The FTIR spectrum of bigel exhibited bands linked to the CO, CW, and AG components. The main bands detected were related to C-H stretching (CH_2_ group) (2917 and 2849 cm^−1^), C=O stretching (ester carbonyl functional group of the triglycerides), C-H of CH_2_ and CH_3_ aliphatic groups (1463 cm^−1^), stretching vibrations of the C-O ester group (1163 cm^−1^), rocking vibrations of -CH_3_ (730 cm^−1^), and C-C (720 cm^−1^), associated with CO and CW. The signals of CH_2_ and CH_3_ aliphatic groups (1463 cm^−1^) were higher in bigel than in butter, indicating a higher concentration of these aliphatic groups in CO-based bigel and reflecting the differences in their lipid compositions, with CO containing more unsaturated fatty acids. The presence of glycosidic linkages (1095–1120 cm^−1^) and -OH stretching vibrations (3392 cm^−1^) due to AG was also observed [13,20,21,22]. In addition, the presence of CW and AG in bigel introduced an additional intensity of the lipid band around 2850 cm^−1^ (C-H stretching) when compared to the butter spectrum.

### 2.2. Quality Characteristics of Cookies

#### 2.2.1. Composition and Amino Acid Profile

The proximate composition of the cookies is listed in Table 2. At the same moisture levels, the protein and ash content were almost equal, whereas the lipid content showed a slight difference: a lipid content of 25.7% for bigel and 23.0% for butter, which could have provided slightly more calories in bigel (504.5 Kcal). These results differ from those of previous studies. The lipid content of cookies made using bigels as fat substitutes tends to be lower than that of cookies made with traditional fats. For example, cookies with sunflower seed powder as a fat replacer had lipid contents ranging from 15.8% to 17.3% [23]. The higher lipid content in bigel cookies could be attributed to the proportion of canola oil used; however, canola oil is a healthier alternative to butter, particularly because of its higher monounsaturated fatty acid and omega-3 fatty acid contents, which are beneficial for health [24]. Significant differences (*p* < 0.05) were found in the amino acid composition; serine, tyrosine, and cysteine contents were lower in bigel cookies, whereas lysine content was lower in butter cookies. Bigel exhibited a lower cysteine value owing to the vegetable source (wheat flour), whereas cookies with commercial butter could incorporate other amino acids from the dairy origin of this fat. Nevertheless, the results demonstrated that the compositional properties of bigel-based cookies were similar to those obtained using butter. This suggests that bigels could be a viable butter substitute for maintaining the nutritional quality of cookies.

#### 2.2.2. Physical Dimensions and Density

**Figure 4:**
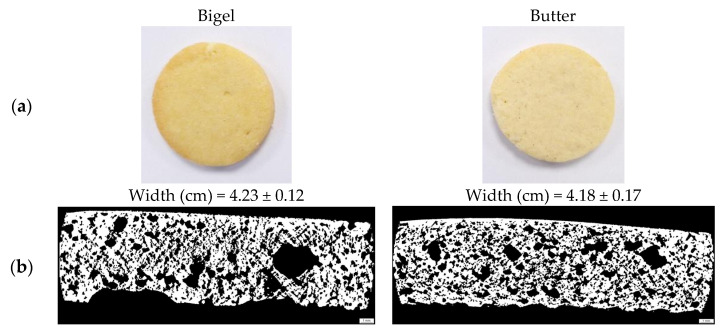
Bottom (**a**) and cross-section (**b**) of baked cookies and their microstructure corresponding cross-sections obtained using micro-CT for bigel (**left**) and butter (**right**) samples. The bar length corresponds to 1000 μm.

Table 3 lists the dimensions of the baked cookies, including their width (W), thickness (T), and spread ratio (W/T). Interestingly, the size and expansion of the cookies did not change significantly (*p* < 0.05) according to the type of fat matter used (Figure 4a). However, the cookies made with bigel had a higher W/T ratio (5.95) than those made with commercial butter (5.54). Chaisawang and Sripywan [25] reported similar spread ratio results when AG was used to replace butter in the cookies. A greater spread ratio is desirable for cookies and biscuits [26], suggesting that bigels have a good potential for use in baking and confectionery preparations. Adding gum typically alters the water absorption and dough development properties, influencing the spread ratio. It has been reported that incorporating AG increases the spread ratio of gluten-free products [27]. This increase could be attributed to the gum in the formulation, which might have caused the oil phase to spread more on the surface of the dough or sugar. This aligns with the textural characterization of fat matter, where bigel spreads more easily than butter. Similarly, a study conducted by Onacik-Gür and Żbikowska [28] observed that cookies with fat with the highest solid phase content had a lower spread ratio. In addition, the spread ratio is inversely proportional to the thickness [29]. In our study, although bigel-based cookies had a reduced thickness (0.71 cm), their spread ratio remained the same, in contrast to butter-based cookies, which exhibited the opposite trend. The true densities of these cookies are listed in Table 3. The values for cookies made with bigel did not differ significantly from those made with butter (*p* < 0.05), confirming that bigels should not cause differences in the actual densities of baked cookies.

#### 2.2.3. Color

The visual characteristics of the top and bottom surfaces of the cookies, as indicated by the BI, revealed that incorporating bigel as a fat source resulted in a slightly higher BI than that of cookies made with butter. In cookies, the observed color development (Figure 4a,b) can be attributed to elevated baking temperatures and a gradual reduction in moisture content, which promotes non-enzymatic browning reactions and caramelization [30]. Other authors found a similar trend for the replacement of butter in cookies using structured oils (e.g., oleogels), indicating that fats with a higher solid fat content (such as butter) bind to and block the substrates of non-enzymatic browning, resulting in the reduction or absence of a reaction. This could explain the differences and reduced BI in cookies made with commercial butter [28,31]. The bottom surface, being exposed to heat and drying first, experienced a reduction in opacity owing to moisture evaporation; however, the temperature increase in this area was insufficient to produce significant variations in the top and bottom browning intensities.

### 2.3. Effect of the Bigel on the Microstructural Properties of Cookies

**Figure 5:**
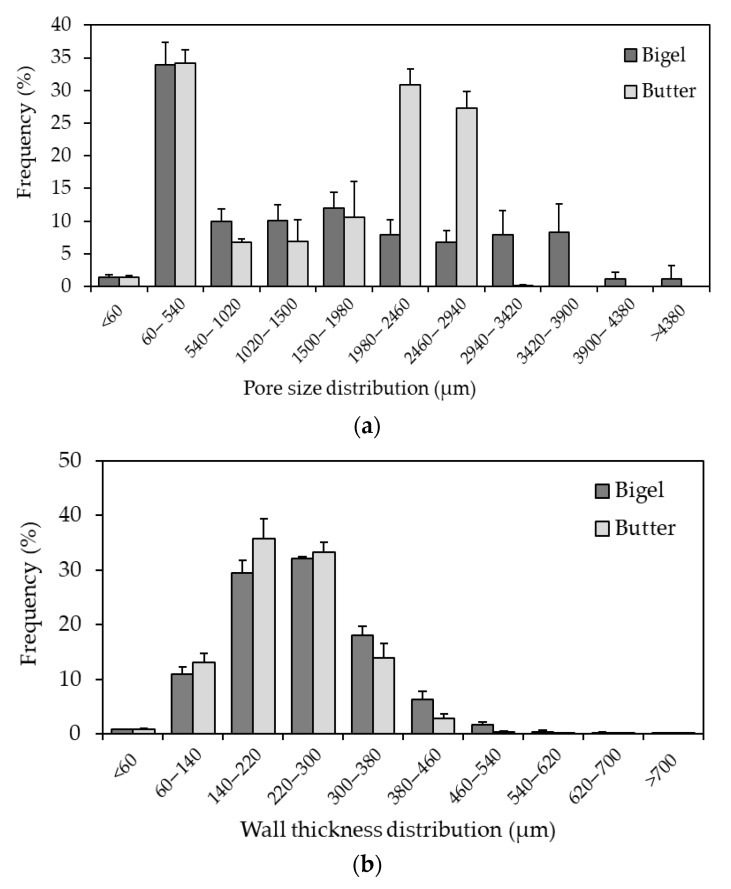
Pore size (**a**) and wall thickness (**b**) distribution of baked cookies obtained with bigel and butter.

The functionality of bakery fats plays a critical role in the product-specific quality characteristics of cookies. Fat influences their macroscopic and microscopic properties [32]. Porosity is a microstructural characteristic closely related to the product volume. Cookies with higher specific volumes tend to have highly porous structures [33], and substituting shortening, margarine, or butter is generally reported to have a negative impact [1]. The microstructures of the baked cookies were analyzed using micro-CT analysis. The impact of replacing butter with bigel on the cookie structure aeration was investigated in terms of porosity, pore size, and wall thickness (Table 3 and Figure 5). Cookie porosity decreased when bigel (55%) was present in the recipe compared to cookies made with butter (61%). In contrast, the mean pore size of bigel-based cookies was significantly higher (1781 µm) (*p* < 0.05) than those made with butter (1051 µm), demonstrating that bigels influence the air incorporated during cookie preparation. Interestingly, the mean wall thickness did not differ significantly (*p* > 0.05) between bigel and butter samples. These findings indicate that cookies made with bigels have structures with large average pore sizes. This may be due to the high thermal stability and good elastic characteristics of bigel during heating (Figure 2d), which affects the expansion mechanism and allows for greater expansion of pores within the baked matrix. These viscoelastic characteristics could reduce cell wall permeability to vapor and slow down temperature-induced permeability changes [34]. In addition, these results are consistent with the previous literature, which has documented that bigels can replicate the functional properties of butter in baking processes by changing the rheological behavior of the matrix, resulting in cookies with comparable internal structures [35]. Thus, the microstructural characteristics of the final product were modulated by the composition of food-grade bigel (canola oil, carnauba wax, and Arabic gum). Kouhsari et al. [36] reported that a possible explanation for the high retention of gases in the structure of biscuits prepared entirely with canola oil, palmitic, and lauric-based fats could be the presence of β’crystals in non-lauric acid-based fats. Martinez-Velasco et al. [37] indicated that candelilla wax/canola oil oleogels favored the retention of a greater amount of gas in sponge cake bread than margarine by stabilizing crystalline films of lipids around the air cells in the matrix. Likewise, Li et al. [31] established that the aeration properties of cookies prepared with wax-based oleogels depend more on β′ crystals.

Previous studies have highlighted that replacing butter with a structured oil, such as an oleogel, in bakery products negatively affects microstructural properties. Using oleogels involves less air being incorporated into the dough, and consequently, a lower total porosity, a smaller pore size, and a denser structure [38,39]. However, bigels can be successfully used as ingredients for total or partial solid fat replacement in complex food matrices and have a comparable cellular structure, demonstrating the importance of fat composition in dough formulation [35]. Figure 5a shows the pore size distributions of both bigel and butter-based cookies. The micro-CT analysis confirmed that cookies made with bigel had a porous structure with larger pores. This result may be explained by the air-holding capacity of the bigel-based dough system, which may lead to a lower gas loss rate and a larger pore size in the baked product, validating the ability of the AG-based bigel to impact the rheology of the cookie batters and suggesting molecular interactions between ingredients. Likewise, other studies have reported that bigels successfully mimic the microstructural arrangement of butter in dough [35,40].

In addition, a detailed distribution of the wall thickness was obtained from the micro-CT analysis of the baked samples (Figure 4b). The structure thickness distribution indicates the mechanical strength of the porous microstructure of the cookies. Although no significant differences were identified in the mean wall thickness between the bigel (254 µm) and butter (244 µm) samples (Table 3), variations were observed in some specific wall-thickness ranges. For example, butter-based cookies exhibited a higher frequency (%) than bigel cookies with wall thickness between 140–220 μm, the highest frequency of all wall thickness ranges analyzed in the two samples. These findings may explain some of the texture results.

### 2.4. Texture Analysis of the Cookies

Hardness is an important cookie quality, along with physical dimensions (width, height, and spread) and color. This texture property strongly depends on the product micro/structure and largely on aeration characteristics [31]. The maximum fracture force is termed hardness. The hardness values of cookies produced using bigel and commercial butter are presented in Table 3. Bigel cookies exhibited significantly (*p* < 0.05) lower hardness values (1.4-fold) than butter cookies did. These results showed an inverse relationship between the mean pore size, thickness, and hardness, probably because larger pores with a similar wall thickness structure resulted in a softer texture than cookies made with butter, which had smaller pores. Similarly, these results indicate that larger heterogeneously distributed air pores provide biscuit products with a lower breaking strength. These morphological characteristics are expected to influence the rate of water transport from the crumb to the crust, thereby modifying the mechanical properties of the cookies. Larger pores in bigel-based cookies are expected to accelerate moisture migration. In contrast, a finer microstructure morphology with smaller gas pores and possibly fewer interconnections between the cells significantly and positively affected the breaking strength in butter cookies. In addition, lower fracturability values (0.70 mm) were observed in bigel cookies than in those made with commercial butter (0.85 mm), indicating that bigel (canola oil/carnauba wax/Arabic gum) promotes baked starch-based matrices with a worsening tendency to deform (or strain) before fracture. Likewise, hard and brittle cookies with reduced expansion have been promoted by commercial butter. Differences in pore size and wall thickness distribution, together with porosity values, may explain the higher hardness and fracturability of the butter cookies, indicating the importance of the morphology of the cellular structure on textural properties and parameters for consumer acceptance and perception, such as crispiness. The presence of larger pores within the cookie matrix can enhance crispness because larger pores reduce the density of the cookie, making it easier to break and providing a more satisfying crunch. Differences in fracturability are directly related to the fatty acid composition of the fat matter [41]. These results demonstrate that fat plays an important role in the microstructure of cookies, which can determine their final textural properties. As bigel properties can be tuned by changing the formulation and processing conditions, further research is needed to optimize bigels to mimic the role of fat in the morphology and microstructure of cookies and the desired texture and crispness.

## 3. Conclusions

Plant-based bigel prepared with a canola oil-carnauba wax oleogel mixed with Arabic gum hydrogel exhibited a softer texture, was more spreadable, and less adhesive than commercial butter. The magnitude of the rheological parameters was lower for bigel than for butter; however, the patterns were similar for both types of fat matter. Bigel exhibited high thermal stability and thixotropic behavior, indicating reversible structural breakdown and recovery. When bigel was used to prepare cookies, a proximate analysis showed similar moisture, protein, and ash content, with a slight increase in lipid content. Cookies prepared with bigel resulted in similar physical dimensions and densities, a slight increase in the browning index, and a decrease in textural parameters, including hardness and fracturability, compared to those prepared with butter. The differences in textural parameters can be explained by the microstructural properties, decrease in porosity, and increase in mean pore size of bigel cookies, which did not affect the mean wall thickness of the samples. The microstructural results can also be explained by the differences between the compositions of food-grade bigel (canola oil, carnauba wax, and Arabic gum) and butter, which impact the porous characteristics of baked cookies. Plant-based bigel prepared with a canola oil-carnauba wax oleogel mixed with Arabic gum hydrogel presented a softer texture, more spreadable, and less adhesive than butter. Cookies prepared with bigel resulted in similar physical dimensions and densities, a slight increase in the browning index, and a decrease in textural parameters, including hardness and fracturability, compared to those prepared with butter. Therefore, incorporating plant-based bigel as a substitute for commercial butter in cookie formulations affected the quality of the formulated cookies because the microstructure changed. However, the magnitude of these changes was low, indicating the potential of Arabic gum-based hydrogels as healthier food ingredients. Bigels offer advantages in terms of temperature stability and microstructural reinforcement, making them a promising alternative to traditional fats, such as butter, in various food applications. Overall, plant-based bigels made from canola oil, carnauba wax, and Arabic gum are promising alternatives to butter from both the economic and sustainability perspectives. Their cost-effectiveness, environmental benefits, and functional properties make them suitable for use in regions where dairy fat is less accessible or more expensive. However, continued efforts to optimize their sensory and nutritional profiles are key to their widespread adoption.

## 4. Materials and Methods

### 4.1. Materials

Cold-pressed canola oil (CO) (7% saturated fatty acids and 88% mono- and polyunsaturated fatty acids) was purchased from Canola de Vida (Osorno, Chile). Carnauba wax (CW) was acquired from Herbolario (Santiago, Chile). Arabic gum (AG) was obtained from Sigma-Aldrich (St. Louis, MO, USA), and commercial butter (53% saturated fatty acids, 25% mono- and polyunsaturated fatty acids, and 3% *trans* fatty acids) was purchased from a local market (Surlat, Loncoche, Chile).

### 4.2. Bigel Preparation

The process conditions to prepare bigel were selected from those reported by Quilaqueo et al. [13]. The oleogel and hydrogel were prepared independently. Subsequently, the two gels were combined and mixed at 60 °C to create a uniform bigel. The oleogel was created by combining 8% (*w*/*w*) CW with CO. The mixture was then heated to 90 °C and stirred at 300 rpm for half an hour. Subsequently, it was allowed to cool to the required temperature. To form the hydrogel, AG was combined with distilled water at a concentration of 4% *w*/*w* and stirred at room temperature until it was dissolved. The bigel was prepared by mixing the oleogel and hydrogel at a 90/10 weight ratio to a total of 100 g. A Benchtop homogenizer (ProScientific, Oxford, CT, USA) was used to blend the two components at 2500 rpm for 7 min. Following preparation, the bigel was placed in glass containers and refrigerated for 24 h before analysis.

### 4.3. Texture and Rheology of Fat Matters

A spreadability test was conducted using a TTS spreadability fixture in a TA.XT PlusC texture analyzer (Stable Micro System, Surrey, UK) and Exponent Connect software version 7.0.6.0 (Stable Micro System, Surrey, UK). Bigel and commercial butter were placed in a conical female probe and stored in a refrigerator at 6 °C for at least 6 h. To conduct the analysis, the male cone penetrated the sample into the female cone at 3 mm/s to a distance of 23 mm and a post-test speed of 10 mm/s. Hardness (maximum force), spreadability (positive area), and adhesiveness (negative area) were determined [8]. At least four replicates were analyzed.

The viscoelastic characteristics of bigel and commercial butter were evaluated using a Discovery DHR2 rheometer (TA Instruments, New Castle, DE, USA) equipped with a parallel rough-plate geometry system (Plate SST ST XHatch 40 mm Smart-SW) and Peltier plate steel, maintaining an axial gap of 1000 μm. This approach was based on the method of Quilaqueo et al. [8] with certain adjustments. To ensure temperature stability, samples were meticulously enclosed in a solvent trap. The TRIOS software suite (version 5.1.1, TA Instruments, New Castle, DE, USA) was used to manage both the equipment operation and rheological data collection. Steady-shear flow measurements were performed at 25 °C, with a shear rate range of 1–1000 s^−1^. The linear viscosity range for each sample was determined by plotting the elastic modulus (G′) against the oscillatory strain (%) under oscillatory conditions at 1 Hz, ranging from 0.01 to 50%. During the frequency sweep test, the temperature was maintained at 25 °C, and the response of the moduli (G′, G″) to increasing frequency (0.1 to 100 Hz) was recorded at a strain of 0.1% within the linear viscosity range. All tests were conducted in triplicate for each sample.

The thermal stability of bigel and butter was evaluated through a temperature sweep test over a temperature range of 20 °C to 90 °C. The analysis was performed at a linear heating rate of 5 °C/min, with a constant shear strain of 0.1% and a frequency of 1 Hz.

The thixotropic properties of bigel and butter were measured by the three-step oscillation method. Three oscillation time sweeps at varying strain amplitudes within and outside the LVR of the sample (400 s at a strain of 0.1%, 200 s at a strain of 100%, and 400 s at a strain of 0.1%) at 25 °C were conducted. Three replicates of each sample were recorded for each test.

### 4.4. Fourier-Transform Infrared (FTIR)

FTIR spectroscopy analyses of the bigel and the butter were performed using an ATR-FTIR Spectrum One spectrometer (PerkinElmer, Waltham, MA, USA). Measurements were conducted at room temperature within a spectral range of 4000 to 400 cm^−1^, with a resolution of 4 cm^−1^ and 32 scans per sample. The acquired spectra were processed using OMNIC 9 software, which included baseline correction and spectral normalization.

### 4.5. Cookie Preparation

Cookies were produced using 500 g wheat flour, 250 g fat (as butter or bigel), 230 g powdered sugar, 5 g NaHCO_3_, and 2 eggs, and were baked at 200 °C, as described by Quilaqueo et al. [8] with a few modifications, until a moisture content of approximately 5% was reached. Three batches were prepared. The cookies were wrapped in paper bags and stored in a dry environment until further analysis.

### 4.6. Nutritional Characterization of Cookies: Proximate Analysis and Amino Acid Profile

The cookie composition was analyzed using the methods outlined by AOAC [42]. The moisture content was measured by oven drying at 105 °C for 24 h (method 945.15), whereas the fat content was assessed by Soxhlet extraction with petroleum ether (method 920.39). Crude protein content was calculated by multiplying the nitrogen content, determined using the Kjeldahl method, by 6.25. The amino acid content was evaluated using reversed-phase high-performance liquid chromatography (RP-HPLC), following the procedure described by Contardo et al. [43]. To determine the caloric intake, the percentage of proteins, carbohydrates, and ether extract in the samples were calculated using Atwater coefficients of 4.0 kcal/g for proteins, 4.0 kcal/g for carbohydrates, and 9.0 kcal/g for lipids [44].

### 4.7. Cookie Texture

To evaluate the effect of the bigel on the texture properties of cookies, hardness and fracturability were determined using a 3-point bending attachment in the TA.XT PlusC texture analyzer (Stable Micro System, Surrey, UK) and Exponent Connect software version 7.0.6.0 (Stable Micro System, Surrey, UK). The samples were compressed at a distance of 10 mm and a speed of 2 mm/s. The maximum force is referred to as hardness, and the distance at the point of the maximum force is related to fracturability [8]. At least six replicates were analyzed.

### 4.8. Expansion and Geometry of Cookies

The thickness and width of the baked cookies were measured using calipers. The spread ratio was determined by dividing the width by the thickness. At least five replicates were analyzed.

### 4.9. Color and Density of Cookies

The surface color variations of the cookies were evaluated at 12 distinct locations using a Minolta Chroma Meter CR-400 (Osaka, Japan). The measurements were analyzed as L* (brightness), a* (red–green spectrum, with positive values indicating redness and negative values indicating greenness), and b* (yellow–blue spectrum, with positive values indicating yellowness and negative values indicating blueness). The browning index (*BI*) was calculated based on the L*, a*, and b* values as follows [45]:

(1) $$BI=\frac{\left[100\times \left(\frac{a^{*}+1.79L^{*}}{5.645L^{*}+a^{*}-3.012b^{*}}-0.31\right)\right]}{0.17}\;\;\;\;.$$

The true density (g/cm^3^) of the cookies was assessed using a nitrogen-operated gas displacement pycnometer (AccuPycII 1340, Micromeritics Instrument Co., Norcross, GA, USA). This measurement was calculated as the weight-to-volume ratio of ground samples. Each test utilized approximately 5–6 g of sample and was conducted three times at room temperature.

### 4.10. X-Ray Microcomputed Tomography (Micro-CT) of Cookies

The cookies were scanned using a high-resolution micro-CT system (SkyScan 1272, version 1.1; Bruker Corp., Kontich, Belgium). The device was operated at a 40 kV source voltage and 250 μA constant source current. Three replicate scans were performed for each sample. The optimal imaging parameters included a 19.9 μm image pixel size, with each image comprising 1344 × 896 pixels. Scans lasted approximately 23 min per sample, covering a 0–360° range at 0.4° rotation steps, with a 0.75 s exposure time per frame and a frame averaging of 2. No filtration was performed. CTAn software (version 1.16.4.1, Skyscan, Belgium) was used for image processing and analysis, following the protocol described by Contardo et al. [46]. This process yielded measurements of the volume occupied by the air-filled pores (total pores) and the solid matrix volume. Air porosity was quantified as the percentage of air-filled pore voxels relative to the total voxel count (encompassing both air and solid matrix within the VOI), with the resulting digital data expressed as a percentage (%).

### 4.11. Statistical Analysis

Statistical analysis involved one-way analysis of variance (ANOVA) to detect significant differences among the samples, with a significance level of 0.05. Tukey’s test was used for further analyses when the ANOVA results indicated significant variations. These statistical procedures were executed using the Statgraphics Centurion XV software (version 15.1.02, The Plains, VA, USA).

## Figures and Tables

**Table 1:** Results of the spreadability test of butter and bigel.

Sample	Firmness (g)	Spreadability (g·s)	Adhesiveness (g)
Butter	29,102 ± 387 ^b^	59,624 ± 2314 ^b^	2282 ± 273 ^b^
Bigel	576 ± 116 ^a^	457 ± 105 ^a^	136 ± 25 ^a^

Different letters in each row indicate significant differences at *p* < 0.05.

**Table 2:** Proximate composition and amino acids profile of cookies prepared with different fat types: bigel or butter.

	Sample	
	Bigel	Butter
**Proximate** **composition**		
Moisture	5.7 ± 0.03 ^a^	5.7 ± 0.03 ^a^
Proteins	7.0 ± 0.02 ^a^	7.2 ± 0.03 ^a^
Lipids	25.7 ± 0.08 ^b^	23.0 ± 0.16 ^a^
Ashes	0.3 ± 0.01 ^a^	0.5 ± 0.00 ^b^
Non-nitrogenous extract	61.3 ± 0.04 ^a^	63.6 ± 0.16 ^b^
Calories (Kcal)	504.5 ± 0.5	490.1 ± 0.2
**Amino acid composition (mg/100 g)**		
Aspartic acid	316.4 ± 1.3 ^a^	296.5 ± 24.9 ^a^
Glutamic acid	2075.0 ± 53.3 ^a^	2179.0 ± 38.8 ^a^
Serine	259.1 ± 1.7 ^a^	326.2 ± 17.0 ^b^
Glycine	208.5 ± 2.7 ^a^	225.9 ± 12.0 ^a^
Histidine	105.6 ± 5.3 ^a^	102.1 ± 16.8 ^a^
Arginine	118.6 ± 7.3 ^a^	127.1 ± 25.0 ^a^
Threonine	131.8 ± 4.2 ^a^	150.3 ± 13.1 ^a^
Alanine	203.0 ± 6.6 ^a^	210.8 ± 15.6 ^a^
Proline	549.7 ± 14.7 ^a^	574.8 ± 33.9 ^a^
Tyrosine	20.1 ± 2.4 ^a^	89.8 ± 13.4 ^b^
Valine	222.6 ± 9.2 ^a^	237.7 ± 12.4 ^a^
Methionine	37.9 ± 3.0 ^a^	40.5 ± 2.1 ^a^
Cysteine	16.5 ± 0.6 ^a^	23.0 ± 1.7 ^b^
Isoleucine	218.5 ± 4.0 ^a^	230.8 ± 0.4 ^a^
Leucine	379.5 ± 6.4 ^a^	392.3 ± 2.9 ^a^
Phenylalanine	241.8 ± 6.4 ^a^	241.1 ± 9.5 ^a^
Lysine	143.7 ± 0.6 ^b^	130.3 ± 3.1 ^a^

Different letters in each row indicate significant differences at *p* < 0.05.

**Table 3:** Characteristics of baked cookies prepared from different fats: butter or bigel.

	Sample	
	Bigel	Butter
**Expansion**		
Width (cm)	4.23 ± 0.12 ^a^	4.18 ± 0.17 ^a^
Thickness (cm)	0.71 ± 0.04 ^a^	0.77 ± 0.09 ^a^
W/T ratio	5.95 ± 0.43 ^a^	5.54 ± 0.71 ^a^
**Density**	1.34 ± 0.00 ^a^	1.37 ± 0.00 ^b^
**Browning index**		
Top	53.42 ± 2.31 ^b^	41.88 ± 0.94 ^a^
Bottom	55.27 ± 2.94 ^b^	47.91 ± 3.75 ^a^
**Texture**		
Hardness (g)	1680 ± 300 ^a^	2350 ± 492 ^b^
Fracturability (mm)	0.70 ± 0.07 ^a^	0.85 ± 0.14 ^b^
**Microstructure**		
Porosity (%)	55 ± 3 ^a^	61 ± 1 ^b^
Mean pore size (µm)	1781 ± 15 ^b^	1051 ± 72 ^a^
Mean wall thickness (µm)	254 ± 19 ^a^	244 ± 15 ^a^

Means with different superscript letters in rows are significantly different (*p* ≤ 0.05) for one-way ANOVA and Tukey’s test.

## Data Availability

The original contributions presented in this study are included in the article. Further inquiries can be directed to the corresponding authors.
